# Onionin A inhibits small-cell lung cancer proliferation through suppressing STAT3 activation induced by macrophages-derived IL-6 and cell–cell interaction with tumor-associated macrophage

**DOI:** 10.1007/s13577-023-00895-6

**Published:** 2023-03-24

**Authors:** Remi Mito, Toyohisa Iriki, Yukio Fujiwara, Cheng Pan, Tsuyoshi Ikeda, Toshihiro Nohara, Makoto Suzuki, Takuro Sakagami, Yoshihiro Komohara

**Affiliations:** 1 Department of Cell Pathology, Graduate School of Medical Sciences, Kumamoto University, Honjo 1-1-1, Kumamoto, 860-8556 Japan; 2 Department of Respiratory Medicine, Graduate School of Medical Sciences, Kumamoto University, Honjo 1-1-1, Kumamoto, 860-8556 Japan; 3 Department of Natural Medicine, Faculty of Pharmaceutical Sciences, Sojo University, Ikeda 4-22-1, Nishi-Ku, Kumamoto, 860-0082 Japan; 4 Department of Thoracic Surgery, Graduate School of Medical Sciences, Kumamoto University, Honjo 1-1-1, Kumamoto, 860-8556 Japan; 5 Center for Metabolic Regulation of Healthy Aging, Kumamoto University, Honjo 1-1-1, Kumamoto, 860-8556 Japan

**Keywords:** Small-cell lung cancer, Interleukin-6, Signal transducer and activator of transcription 3, Tumor-associated macrophage, Cell–cell interaction

## Abstract

Tumor-associated macrophage (TAM)-derived IL-6 is involved in small-cell lung cancer (SCLC) progression and chemoresistance via the activation of signal transducer and activator of transcription 3 (STAT3) in the tumor microenvironment. This study aimed to identify natural compounds that suppress cell–cell interactions between TAMs and SCLC cells by inhibiting STAT3 activation. We used a library of natural compounds to identify candidate agents possessing anti-SCLC effects by inhibiting macrophage-induced tumor proliferation. SBC-3 and SBC-5, human SCLC cell lines, were used for in vitro experiments. Furthermore, we assessed the efficacy of these candidate agents in a murine xenograft model of human SCLC. Among the natural compounds examined, onionin A (ONA) inhibited IL-6-induced STAT3 activation and SCLC cell proliferation. ONA also reduced the secretion of IL-6 from macrophages and interfered with the direct effect of cell–cell interactions between macrophages and SCLC cells. Furthermore, ONA administration suppressed tumor progression in a tumor-bearing mouse model. ONA was identified as the most useful candidate for targeting cell–cell interactions between cancer cells and TAMs for anti-SCLC therapy.

**Supplementary Information:**

The online version contains supplementary material available at 10.1007/s13577-023-00895-6.

## Introduction

Lung cancer is the most common cause of cancer-related death worldwide. Oncogenic driver mutations have received the most attention in non-small cell lung cancer (NSCLC) research, and personalized therapies based on these genetic analyses have recently been developed for their treatment. [1–4]. Immuno-checkpoint inhibitors radically alter the treatment of lung cancer, resulting in a marked improvement in the prognosis of NSCLC [5, 6]. However, the effects of immune checkpoint inhibitors are limited for small cell lung cancer (SCLC), and the number of patients who can undergo surgery is very limited because more than 95% of patients with SCLC are diagnosed at advanced stages [7]. Therefore, the prognosis of SCLC remains poor. Recently, SCLC has been classified into four phenotypes according to the expression levels of transcription factors such as *ASCL1*, *NEUROD1*, *YAP1*, and *POU2F3* [8, 9]. Clinical trials targeting these subtypes have been conducted [10, 11]; however, SCLC, a highly aggressive cancer that represents 14–20% of all lung cancers, has not seen major therapeutic advances in the past 30 years [12–14] and the identification of novel therapeutic target molecules is needed.

Signal transducer and activator of transcription 3 (STAT3) is one of the most critical signal molecules in tumor cells. In several malignant tumors, STAT3 activation is related to tumor metastasis, invasion, proliferation, and acquisition of tolerance to chemotherapy and radiotherapy [15, 16]. We previously revealed that TAMs support tumor progression through STAT3 activation in several malignant tumors [17–20]. In NSCLC, persistent STAT3 activation is present in 22–65% of cases, and the activation of STAT3 is involved in tumor proliferation, chemotherapy tolerance, and poor clinical outcome [21–24]., STAT3 activation is observed in every case of SCLC [25], suggesting that STAT3 activation plays a very important role in SCLC progression. Nevertheless, few studies have examined the relationship between tumor progression and STAT3 activation in SCLC. Infiltrating macrophages (TAMs) with the M2 phenotype [26–28] have been observed in lung cancer and play a critical role in the angiogenesis, invasion, progression, metastasis, immunosuppression, and matrix remodeling that are involved in cancer [29, 30]. CD163 is a hemoglobin scavenger receptor expressed in macrophages and is widely used as a marker for the M2-like/pro-tumor phenotype of TAMs [31]. Although the significance of CD163 in SCLC is unknown, many studies have demonstrated that CD163-positive TAMs are associated with a worse clinical course in many cancers [32]. Studies using animal models have shown that CD163-positive macrophages have more pro-tumor functions than CD163-negative macrophages [33] and that CD163-mediated macrophage activation is linked to the secretion of pro-tumor cytokines, such as interleukin (IL)-6 and chemokine (C-X-C motif) ligand 2 (CXCL2) [34]. Therefore, CD163-positive TAMs are considered targets for antitumor therapy. We previously revealed that onionin A (ONA), a cyclic sulfur-containing natural compound isolated from onion, exerts an inhibitory effect on the progression of certain tumors, such as glioblastoma, osteosarcoma, and ovarian cancer, by changing the M2 phenotype into the M1 phenotype in macrophages via CD163 inhibition [20, 35, 36]. Conversely, STAT3 activation is significantly enhanced by stimulation of macrophage-derived culture supernatant (CS) in SCLC cells, and macrophage-derived factors such as CCL4 and IL-6 are involved in the activation of STAT3 in SCLC cells [37], suggesting that TAMs probably participate in SCLC progression via STAT3 activation. Thus, the cell–cell interaction between SCLC cells and TAMs could be a target for anti-SCLC treatment. In this study, we aimed to identify candidate natural compounds with anti-SCLC effects.

## Materials and methods

### Immunohistochemistry

Paraffin-embedded tissue samples of primary lesions from 14 patients with SCLC who underwent surgery at the Kumamoto University Hospital between January 2004 and September 2014 were used. The patients provided written informed consent in accordance with the protocols of the Review Board in Kumamoto University (#2224). Samples were sectioned into 3-µM thick specimens and embedded in paraffin for immunostaining. Anti-phosphorylated STAT3 (pSTAT3) antibody (D3A7, Cell Signaling Technology Japan, Tokyo, Japan) and anti-CD163 antibody (10D6, Leica Biosystems, Nussloch, Germany) were used as primary antibodies, and horseradish peroxidase-labeled anti-mouse or anti-rabbit immunoglobulin antibodies (Nichirei, Tokyo, Japan) were used as secondary antibodies. A DAB substrate system (Nichirei, Tokyo, Japan) was used to visualize the immunoreactions. Double immunostaining was performed to identify the colocalization of pSTAT3 and CD163. First, the sections were stained with anti-pSTAT3 and visualized using DAB, as described above. The anti-CD163 antibody was then used and visualized using HistoGreen (Eurobio Scientific).

### Cells and cell culture conditions

The human SCLC cell lines SBC-3 and SBC-5 were purchased from the JCRB Cell Bank (Osaka, Japan). The cells were maintained in D-MEM/Ham’s F-12 medium supplemented with 10% fetal bovine serum (FBS) (Sigma-Aldrich, St. Louis, MO, USA) in a 37 °C incubator with 5% CO_2_. The cultures were regularly tested and were found to be negative for *mycoplasma* contamination.

Peripheral blood mononuclear cells (PBMCs) were obtained from healthy volunteers. Written informed consent for blood sampling and subsequent analysis was obtained from all the healthy volunteers. All protocols using human samples were approved by the Kumamoto University Hospital Review Board (No. 486) and performed according to approved guidelines. Via positive selection using magnetic-activated cell sorting (Miltenyi Biotec, Bergisch Gladbach, Germany), CD14^+^ monocytes were refined from peripheral blood mononuclear cells. CD14^+^ monocytes were cultured in AIM-V medium (Gibco, USA) containing 10% FBS and 10 ng/ml GM-CSF (Wako, Tokyo, Japan) or 100 ng/ml M-CSF (WAKO) for seven days to differentiate into macrophages. The differentiated macrophages were used as human monocyte-derived macrophages (HMDMs) as described previously [37]. Although M-CSF induces M2 phenotype differentiation and GM-CSF induces M1 phenotype differentiation in macrophages [37], macrophage-derived culture supernatant (CS) was prepared from both phenotypes and showed similar responses in SCLC cell lines.

Primary murine peritoneal macrophages were obtained from the peritoneal exudate fluid of mice. Peritoneal macrophages were cultured in D-MEM/Ham’s F-12 medium supplemented with 10% FBS, penicillin G 100U/ml and streptomycin 100 μg/ml.

### Preparation of macrophages and lymphocyte culture supernatants

Human monocyte-derived macrophages and mouse peripheral macrophages were maintained in D-MEM/Ham’s F-12 medium supplemented with 10% FBS, penicillin G 100U/ml and streptomycin 100 μg/ml for 24 h. Macrophage-derived culture supernatants (CS) were centrifuged at 178 × g for 10 min. Lymphocytes were cultured in D-MEM/Ham’s F-12 medium supplemented with 10% FBS, penicillin G 100U/ml and streptomycin 100 μg/ml for 5 days, and then lymphocyte-derived CS was centrifuged at 178 × g for 10 min.

### STAT3 activation assay

STAT3 activation was assessed by measuring the increased expression of pSTAT3 by western blotting, as previously described [37]. Solubilized SBC-3 cells and macrophages were run on a 10% SDS–polyacrylamide gel and transferred to a polyvinylidene fluoride (PVDF) transfer membrane (Millipore, Bedford, MA, USA). To detect pSTAT3, the membranes were incubated with an anti-pSTAT3 antibody (D3A7; Cell Signaling Technology Japan, Tokyo, Japan) and visualized using a horseradish peroxidase-conjugated anti-rabbit IgG antibody with ECL western blotting detection reagent (GE Healthcare Life Sciences, Piscataway, NJ, USA). To detect STAT3, the membranes were incubated with an anti-STAT3 antibody (124H6; Cell Signaling Technology Japan, Tokyo, Japan) and visualized using a horseradish peroxidase-conjugated anti-mouse IgG antibody with an ECL western blotting detection reagent. The membranes were re-blotted with anti-β-actin antibody (C4) (sc-47778; Santa Cruz Biotechnology, Inc.) as an internal calibration control. Quantification of the western blots was performed using ImageJ and the Amersham Imager 680 analysis software.

### Cell proliferation and cytotoxicity assays

SBC-3 (1 × 10^3^ cells/well) and SBC-5 cells (1 × 10^3^ cells/well) were cultured in a 96-well plate in quadruplicate prior to ONA treatment. In the co-culture model, SBC-3 (1 × 10^3^ cells/well) and SBC-5 cells (1 × 10^3^ cells/well) were cultured with macrophages (3 × 10^3^ cells/well) in a 96-well plate. Cell viability and proliferation of tumor cells were detected using the WST assay (WST-8 Cell Counting Kit; Doujin Chemical, Kumamoto, Japan) and BrdU ELISA Kit (Roche, Penzberg, Bavaria, Germany), respectively, following the manufacturer’s protocols.

### Indirect co-culture using a cell culture insert

SBC-3 cells (3 × 10^4^ cells/well) were cultured on the bottom plate of a 6-well (Multiwell 6-well plate), and macrophages (1 × 10^5^ cells/well) were plated on a 6-well cell culture insert in quadruplicate prior to ONA treatment. After 24 h of incubation, SBC-3 cells were collected to assess the expression of phosphorylated STAT3 and STAT3 by western blot analysis.

### Cell block preparation

SBC-3 cells were cultured in a 10 cm culture dish in D-MEM/Ham’s F-12 medium supplemented with 10% FBS in a 37° C incubator with 5% CO_2_ and collected in a 50 ml tube. After centrifugation at 400 × g for 5 min, the supernatant was removed, leaving the cell pellet, and mixed with 3 ml of 10% formalin and 10 μl of eosin. After allowing to stand at room temperature for 3 h, it was centrifuged at 400 × g for 5 min, the supernatant was removed, and mixed with 1% sodium alginate (0.5 mL), followed by removal of the supernatant and mixing with 100 μl of 1 M calcium chloride. The agarose cell pellet was then removed and embedded in paraffin. Paraffin-embedded cell blocks were used for the immunostaining of pSTAT3.

### Quantification of immunostaining images

Cell blocks of SBC-3 cells and tumors resected from murine xenograft models were sectioned into 3-µM thick specimens and immunostained for pSTAT3. Five images were obtained using a bright-field microscope with a 20 × objective lens. The percentage of pSTAT3-positive cells was measured using the ImageJ Fiji software.

### Recombinant proteins and neutralizing antibody

Recombinant human IL-6 was obtained from Wako Pure Chemical Industries (Osaka, Japan). Anti-human IL-6 receptor (IL-6R) neutralizing monoclonal antibody was purchased from Absolute Antibody Ltd. (Redcar, UK).

### Natural compounds

Corosolic, glycyrrhizic, oleanolic, and betulinic acids were purchased from Fujifilm Wako (Osaka, Japan). Oleanonic acid was purchased from Selleck Biotech (Tokyo, Japan). Onionin A was prepared as previously described [38].

### Enzyme-linked immune-sorbent assay

Human monocyte-derived macrophages and lymphocytes were incubated with SBC-3 cell culture supernatants (CS) for 24 h, and the IL-6 concentration in the CS was measured using an IL-6 ELISA kit (BioLegend, San Diego, CA) according to the manufacturer’s protocols.

### Animal experiments

Male BALB/cAJc1-nu/nu mice (4–6 w) were purchased from Charles River Laboratories (Yokohama, Japan). SBC-3 cells (2.0 × 10^6^) were suspended in 100 µL of PBS and subcutaneously injected into the right and left flanks of the mice. After tumor grafting was confirmed, mice were randomly assigned to two groups (*n* = 13). Mice were treated with 20 mg/kg ONA by intraperitoneal injection every 2 days for a total of 14 days (seven doses), followed by determination of subcutaneous tumor development. All animal experiments were approved by the Ethics Committee for Animal Experiments of Kumamoto University (Permission Number: B24-125) and conducted in accordance with the Guidelines for Animal Experiments of Kumamoto University.

### Statistics

All data are representative of two or three independent experiments. Data are expressed as mean ± standard deviation (SD). Statistically significant differences between groups were examined using the Mann–Whitney U-test and one-way ANOVA. Statistical significance was set at *P* < 0.05.

## Results

### STAT3 activation in cancer cells is potentially linked to CD163-positive TAM-related signals

**Fig. 1:**
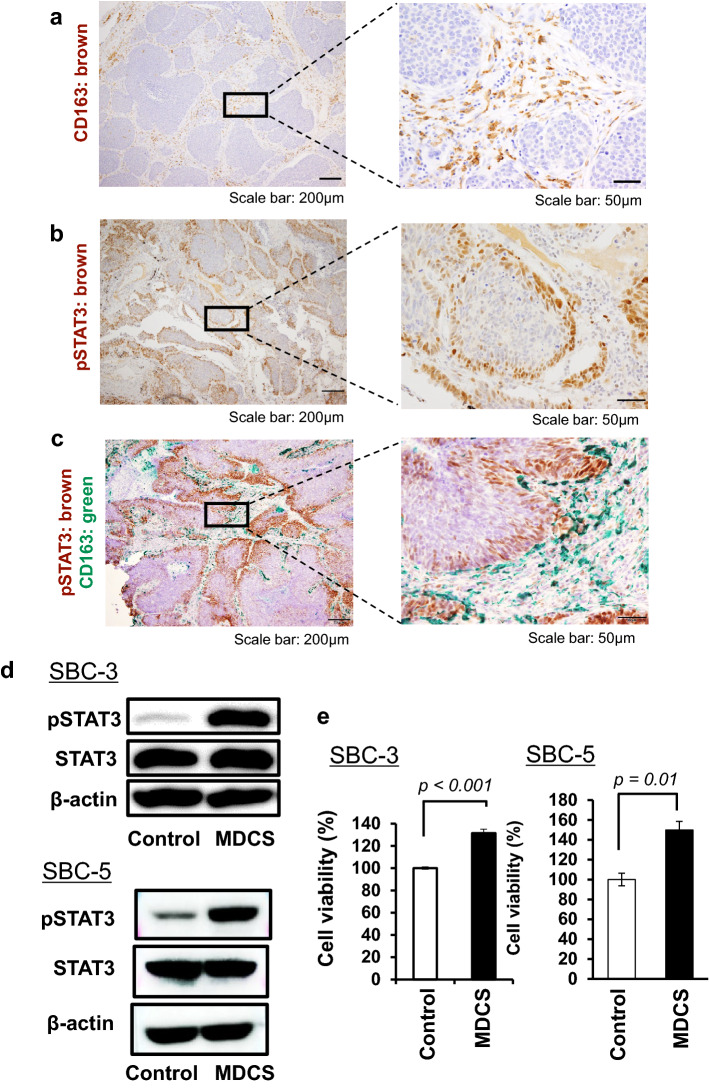
The activation of STAT3 in SCLC tissues and SBC-3 cells. Immunohistochemical staining for the **a** macrophage marker CD163 and **b** phosphorylated STAT3 in surgically resected SCLC tissues. **c** Double immunohistochemical staining of phosphorylated STAT3 (brown) and CD163 (green) in surgically resected SCLC tissues. **d** SBC-3 cells and SBC-5 cells stimulated with macrophage-derived culture supernatant (MDCS) for 24 h and assessed for expression of phosphorylated STAT3, and STAT3 by western blotting analysis. β-actin expression was used as an internal control. **e** SBC-3 cells and SBC-5 cells stimulated with MDCS for 48 h and assessed for cancer cell proliferation using the WST-8 assay

We first investigated the histological correlation between CD163^+^ TAMs and cancer cells using paraffin-embedded tissue samples from patients with SCLC. Numerous CD163^+^ TAMs were observed in the stromal area; however, TAMs were rarely detected in cancer nests (Fig. 1a). Phosphorylated STAT3 (pSTAT3) was detected in the nuclei of cancer cells, and a higher positive signal was observed in the peripheral area than in the central area of the cancer nest (Fig. 1b), which is consistent with our previous study [35]. Double immunohistochemical (IHC) analysis of CD163 and pSTAT3 expression showed that STAT3 activation was induced in cancer cells located near TAMs (Fig. 1c). Similar observations were made for all SCLC samples.

Next, in vitro studies were performed to test cell–cell interactions between SCLC cells and human monocyte-derived macrophages. As shown in Fig. 1d, macrophage-derived culture supernatant (MDCS) enhanced STAT3 activation in SBC-3 and SBC-5 cells. MDCS also enhanced tumor proliferation in both tumor cell lines (Fig. 1e), indicating that MDCS is correlated with tumor proliferation via STAT3 activation in SCLC cells.

### Onionin A (ONA) inhibits MDCS-induced STAT3 activation in SCLC cells

**Fig. 2:**
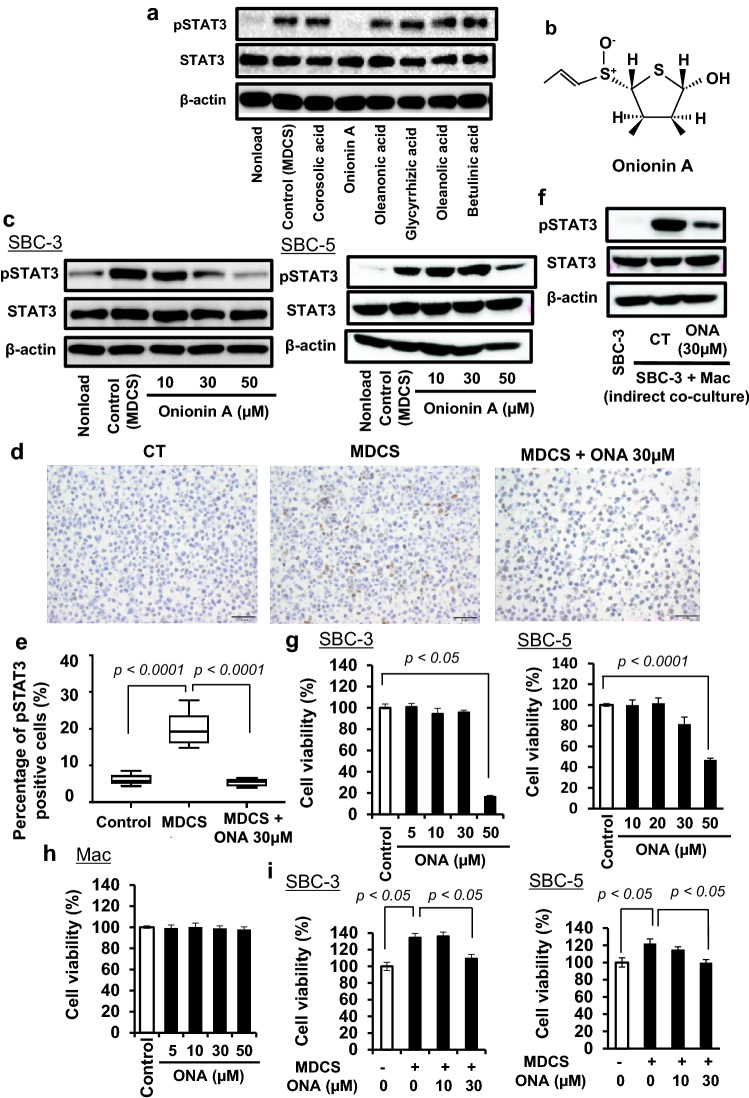
Onionin A can suppress macrophage-derived CS-induced STAT 3 activation and proliferation in SBC-3 cells **a** SBC-3 cells stimulated with MDCS in the presence of natural compounds (50 µM) for 24 h, followed by measurement of phosphorylated STAT3 (pSTAT3), and STAT3 expression by western blotting analysis. β-actin expression was used as a control. **b** Chemical structure of onionin A. **c** SBC-3 cells and SBC-5 cells stimulated with MDCS in the presence of onionin A for 24 h, and assessed for pSTAT3, and STAT3 expression using western blotting analysis. β-actin expression was used as an internal control. **d** SBC-3 cells stimulated with MDCS in the presence of onionin A for 48 h, and prepared cell blocks, followed by determination of phosphorylated STAT3 expression by immunostaining. **e** Percentage of pSTAT3 positive cells analyzed, respectively. **f** SBC-3 cells cultured with macrophages under insert co-culture systems in the presence of onionin A (30 µM) for 24 h, and assessed for pSTAT3, and STAT3 expression using western blotting analysis. β-actin expression was used as an internal control. **g** SBC-3 cells stimulated with onionin A (ONA) and assessed for cell viability using a WST-8 assay. **h** Human monocyte-derived macrophages stimulated with ONA and assessed for cell viability using a WST-8 assay. **i** SBC-3 cells stimulated with ONA in the presence of MDCS for 24 h, followed by the assessment of cancer cell proliferation using a WST-8 assay

We investigated the effects of natural compounds on MDCS-induced STAT3 activation in SBC-3 cells to identify candidate agents for the inhibition of macrophage-induced cancer proliferation. A previous study showed that the natural compounds used in the present study inhibited IL-10- or IL-6-induced STAT3 activation in macrophages [39–44]; however, the effects of these natural compounds on MDCS-induced STAT3 activation in SCLC cells are unknown. Our study showed that among these natural compounds, only ONA significantly inhibited MDCS-induced STAT3 activation in SBC-3 and SBC-5 cells (Fig. 2a, b) and exerted an inhibitory effect on MDCS-induced STAT3 activation in a dose-dependent manner (Fig. 2c). Similar results were also observed using IHC (Fig. 2d and e). Furthermore, ONA suppressed STAT3 activation in SBC-3 cells under indirect co-culture conditions with macrophages using a cell culture insert (Fig. 2f), suggesting that ONA suppresses the effect of macrophage-derived soluble factors, which induce STAT3 activation in SCLC cells.

### ONA inhibits MDCS-induced SCLC cell proliferation

We investigated the effect of ONA on the proliferation of SBC-3 and SBC-5 cells. First, we assessed the direct inhibitory effect of ONA on proliferation in both tumors. ONA significantly inhibited tumor proliferation in both tumor cell lines at 50 µM (Fig. 2g), whereas it had no cytotoxic effect on human monocyte-derived macrophages at concentrations up to 50 µM (Fig. 2h). In contrast, 30 µM ONA inhibited MDCS-induced proliferation in both tumor cell lines (Fig. 2i). These results suggest that ONA suppresses macrophage-derived soluble factors, which induce cancer cell proliferation.

### IL-6 is suggested as one of protumor cytokines in MDCS

**Fig. 3:**
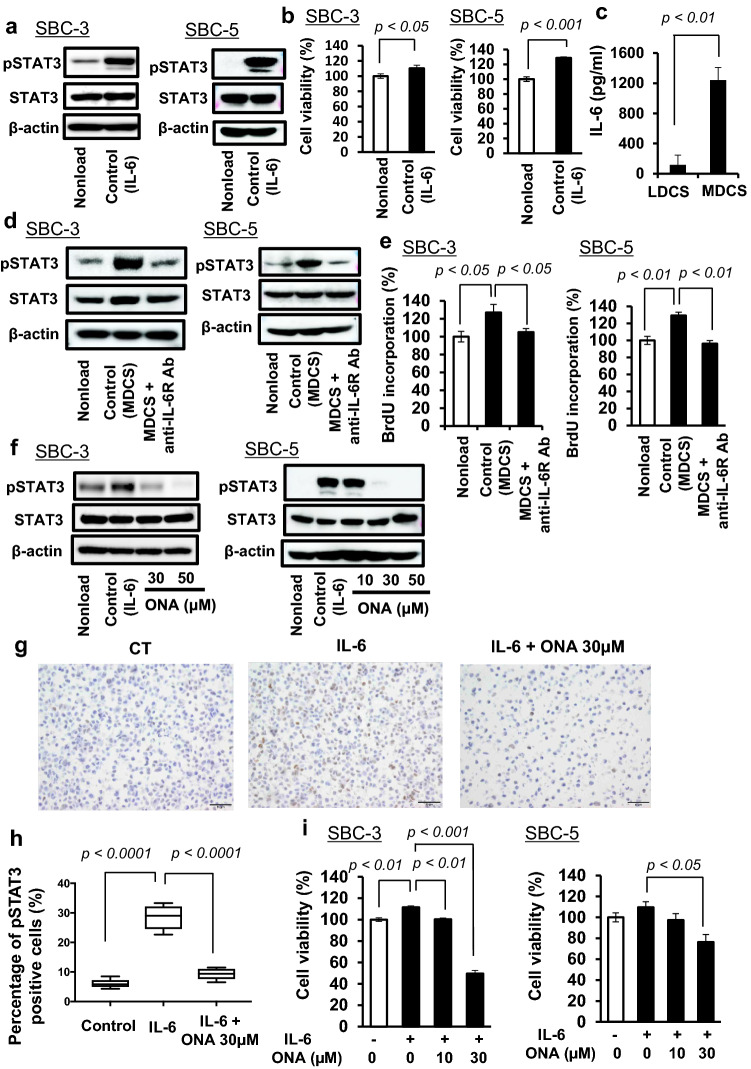
Onionin A inhibits macrophage-derived IL-6-induced STAT3 activation and tumor proliferation. **a** SBC-3 cells and SBC-5 cells stimulated with IL-6 for 24 h, followed by measurement of pSTAT3, and STAT3 expression by western blotting analysis. β-actin expression was used as a control. **b** SBC-3 cells and SBC-5 cells stimulated with IL-6 for 48 h, followed by the determination of cancer cell proliferation using a BrdU assay kit. **c** Human monocyte-derived macrophages and human lymphocytes incubated for 48 h, followed by the measurement of IL-6 concentration in the macrophage-derived culture supernatant (MDCS) and lymphocyte-derived culture supernatant (LDCS) by ELISA. **d** SBC-3 cells and SBC-5 cells stimulated with MDCS in the presence of anti-human IL-6 receptor (IL-6R) neutralizing antibody for 48 h, and then assessed for pSTAT3, and STAT3 expression using western blotting analysis. β-actin expression was used as an internal control. **e** SBC-3 cells and SBC-5 cells incubated with MDCS in the presence of anti-human IL-6R neutralizing antibody for 48 h, followed by cancer cell proliferation measurement using the BrdU assay. **f** SBC-3 cells and SBC-5 cells stimulated with IL-6 in the presence of onionin A for 48 h, followed by the evaluation of pSTAT3, and STAT3 expression using western blot analysis. β-actin expression was used as an internal control. **g** SBC-3 cells stimulated with IL-6 in the presence of onionin A for 48 h, and prepared cell blocks, followed by determination of phosphorylated STAT3 expression by immunostaining. **h** Percentage of pSTAT3 positive cells analyzed, respectively. **i** SBC-3 cells and SBC-5 cells stimulated with IL-6 in the presence of onionin A for 48 h, followed by cancer cell proliferation measurement using a WST-8 assay

It has previously been reported that IL-6, a soluble factor present in MDCS, enhances tumor proliferation in SBC-3 cells [37]. We found that IL-6 induced both STAT3 activation and tumor proliferation in SBC-3 and SBC-5 cells using western blotting analysis and a WST-8 assay (Fig. 3a and b). In addition, IL-6 was detected in MDCS (Fig. 3c), and the IL-6R-neutralizing antibody inhibited both STAT3 activation and tumor proliferation induced by MDCS stimulation in both tumor cell lines (Fig. 3d and e). These results suggest that macrophage-derived soluble factors, especially IL-6, induce STAT3 activation and tumor proliferation in SBC-3 and SBC-5 cells.

### ONA inhibited IL-6-induced SCLC proliferation

Next, we tested whether ONA affects IL-6-induced STAT3 activation and proliferation in SBC-3 and SBC-5 cells. We found that ONA inhibited IL-6-induced STAT3 activation in SBC-3 and SBC-5 cells (Fig. 3f). These results were also confirmed by IHC using cell blocks, with statistically significant differences (Fig. 3g and h). In addition, ONA suppressed IL-6-induced SBC-3 and SBC-5 proliferation (Fig. 3i), suggesting that ONA prevents macrophage-derived soluble factors, including IL-6, from inducing tumor proliferation.

### ONA inhibits SCLC proliferation by suppressing macrophage IL-6 secretion and regulating tumor proliferation induced by direct cell–cell contact with macrophages

**Fig. 4:**
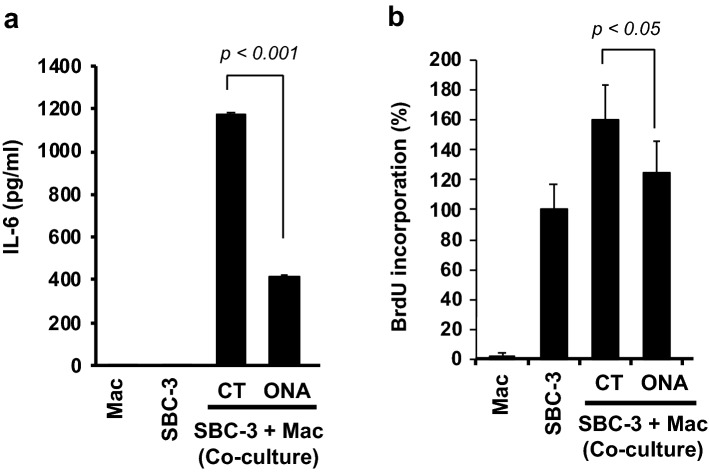
The effect of onionin A on IL-6 production and tumor proliferation in SCLC cells co-cultured with macrophages. **a** SBC-3 cells and human monocyte-derived macrophages (HMDMs) stimulated with 30 µM onionin A under co-culture condition for 48 h, followed by the measurements of IL-6 production in the culture supernatant by ELISA. **b** SBC-3 cells and HMDMs stimulated with 10 µM onionin A for 48 h under co-culture conditions, followed by cancer cell proliferation assessment using a BrdU assay

ONA significantly suppressed IL-6 production under coculture conditions with macrophages and SBC-3 cells (Fig. 4a). Furthermore, we previously reported that direct cell–cell interactions between macrophages and SCLC cells enhanced strong tumor proliferation [37], and ONA also suppressed SBC-3 proliferation enhanced by cell–cell interactions with macrophages under co-culture conditions at 10 µM (Fig. 4b). This concentration was lower than that of ONA, which inhibited MDCS-induced SCLC cell proliferation. Therefore, these results suggest that ONA could inhibit SBC-3 proliferation by suppressing both indirect and direct cell–cell interactions between macrophages and SCLC cells.

### ONA inhibits cell–cell interaction between mouse macrophages and SCLC cells

**Fig. 5:**
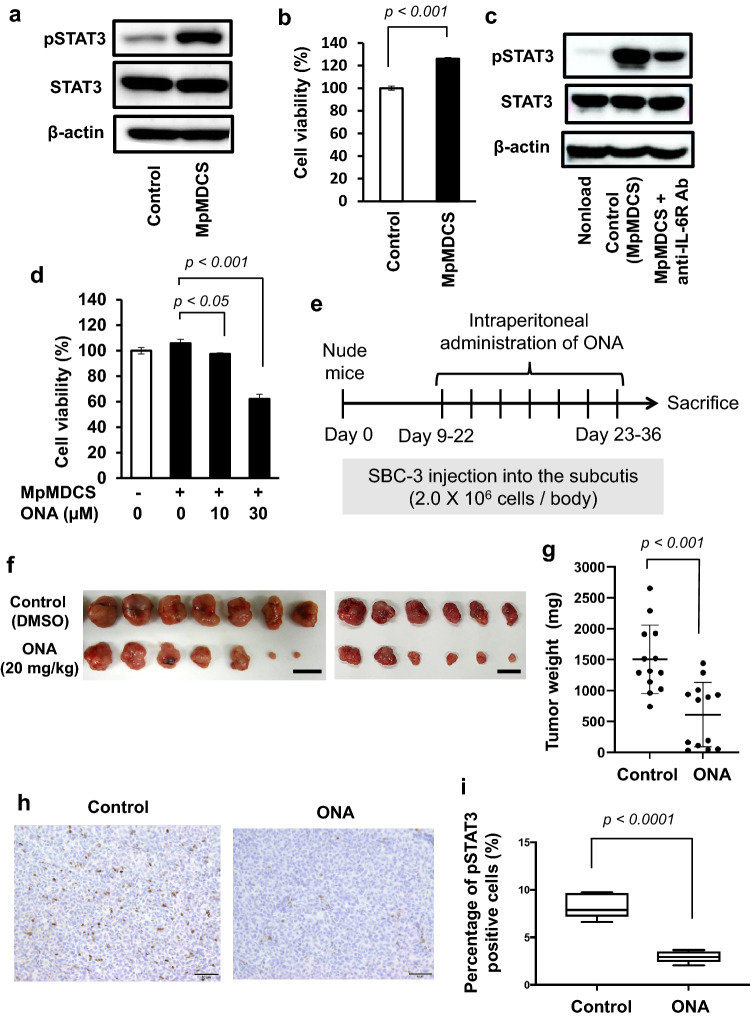
Onionin A inhibits mouse peritoneal macrophages-derived CS-induced STAT 3 activation and proliferation in SBC-3 cells and suppresses SCLC development in a murine xenograft model. **a** SBC-3 cells stimulated with mouse peritoneal macrophages-derived culture supernatant (MpMDCS) for 24 h, followed by the evaluation of pSTAT3 and STAT3 expression using western blot analysis. β-actin expression was used as an internal control. **b** SBC-3 cells were stimulated with MpMDCS for 48 h. Then cancer cell proliferation was measured using a WST-8 assay. **c** SBC-3 cells stimulated with MpMDCS in the presence of anti-human IL-6 receptor (IL-6R) neutralizing antibody for 48 h, and then assessed for pSTAT3, and STAT3 expression using western blotting analysis. β-actin expression was used as an internal control.** d** SBC-3 cells stimulated with ONA in the presence of MpMDCS for 48 h, followed by cancer cell proliferation measurement using a WST-8 assay. **e** The protocol for onionin A administration and SBC-3 cell implantation in nude mice. **f** Nude mice (*n* = 13) were subcutaneously injected with SBC-3 cells and administered 20 mg/kg onionin A by intraperitoneal injection every 2 d for a total of 14 d (e.g., seven doses). **g** Tumor weights analysis. **h** Phosphorylated STAT3 expression in tumor tissue determined using IHC. **i** Percentage of pSTAT3 positive cells analyzed, respectively

We tested the anti-SCLC effects of ONA using a murine model by examining cell–cell interactions between mouse macrophages and SBC-3 cells. Similar to human MDCS, mouse peritoneal macrophage-derived CS (MpMDCS) enhanced STAT3 activation and SBC-3 cell proliferation in vitro (Fig. 5a and b). Furthermore, the IL-6R-neutralizing antibody inhibited STAT3 activation induced by MpMDCS stimulation in SBC-3 cells (Fig. 5c), suggesting that MpMDCS also enhances tumor proliferation via STAT3 activation induced by the IL-6/IL-6R signaling pathway. In addition, ONA also inhibited the MpMDCS-induced proliferation of SBC3 cells (Fig. 5d), indicating that ONA inhibits cell–cell communication between mouse macrophages and SCLC cells.

### ONA suppressed SCLC development in murine model

The anti-cancer effects of ONA were tested in an SBC-3-bearing mouse model. ONA was administered intraperitoneally (Fig. 5e) and the results showed that ONA administration significantly inhibited SBC-3 development in vivo (Fig. 5f and g).

ONA administration also suppressed STAT3 activation in the tumor tissues (Figs. 5h and i)*.* Furthermore, no adverse events such as hepatic injury, renal toxicity, or lung injury were observed in the blood tests (Fig. S1). These results indicate that ONA may be an advantageous treatment option for SCLC.

## Discussion

**Fig. 6:**
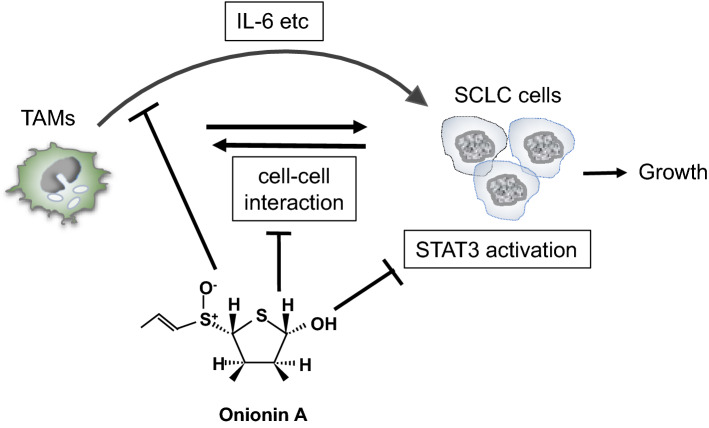
Schema of a potential anti-SCLC model of ONA. Onionin A inhibited IL-6 production by macrophages and macrophages-derived IL-6 induced STAT 3 activation. Onionin A also inhibited direct cell–cell interactions between macrophages and SCLC cells. These functions of onionin A contributed to the suppression of tumor progression in SCLC cells

We demonstrated that STAT3 activation was preferentially observed in SCLC cells adjacent to the stromal area, where many infiltrated CD163^+^ TAMs were observed. STAT3 is a recognized signaling molecule related to cancer cell growth, chemoresistance, invasion, and angiogenesis in many types of cancers. The results of this study suggest that macrophage-derived factors, including IL-6, activate STAT3 signaling and proliferation in SCLC cells. In this study, ONA was found to have an anti-SCLC effect in in vitro cell culture and in vivo murine models using SBC-3 and SBC-5 cell lines. To our knowledge, this is the first study to report the anti-SCLC effects of ONA. We have previously reported that ONA inhibits tumor progression in sarcoma and ovarian cancers by regulating macrophage activation [36, 40]; however, this is the first evidence that ONA can inhibit STAT 3 activation induced by IL-6, which was derived from macrophages in SCLC cells. Furthermore, onionin A inhibited direct cell–cell interactions between macrophages and SCLC cells and suppressed IL-6 production by macrophages. Therefore, onionin A is a promising treatment for SCLC that can suppress both indirect and direct cell–cell interactions between macrophages and SCLC cells (Fig. 6). Nevertheless, the mechanism by which ONA prevents cell–cell interactions between TAMs and SCLC cells remains unknown and further studies are required.

## Conclusion

The present study revealed that ONA suppressed SCLC growth by inhibiting STAT3 activation induced by TAM-derived factors including IL-6. Thus, ONA may be an advantageous treatment for patients with SCLC.

## Supplementary Information

Below is the link to the electronic supplementary material.

Supplementary file1 (PPTX 5054 KB)

## Data Availability

The data in this research are available upon request from the corresponding author.

## Abbreviations

SCLC: Small-cell lung cancer
NSCLC: Non-small cell lung cancer
TAM: Tumor-associated macrophage
HMDMs: Human monocyte-derived macrophages
ONA: Onionin A
STAT3: Signal transducer and activator of transcription 3
pSTAT3: Phosphorylated STAT3
IL-6: Interleukin-6
IL-6R: IL-6 receptor
CXCL2: Chemokine (C-X-C motif) ligand 2
CS: Culture supernatant
MDCS: Macrophage-derived culture supernatant
MpMDCS: Mouse peritoneal macrophage-derived CS
FBS: Fetal bovine serum
IHC: Immunohistochemical
PVDF: Polyvinylidene fluoride
SD: Standard deviation
