# Supplementary material for: Onionin A inhibits small-cell lung cancer proliferation through suppressing STAT3 activation induced by macrophages-derived IL-6 and cell–cell interaction with tumor-associated macrophage
Source: Hum Cell. 2023 Mar 24;36(3):1068–80. doi: 10.1007/s13577-023-00895-6 (PMC10110690; doi:10.1007/s13577-023-00895-6)
Supplement: Supplementary file 1 — Supplementary file1 (PPTX 5054 KB) [file 13577_2023_895_MOESM1_ESM.pptx]

## Slide 1
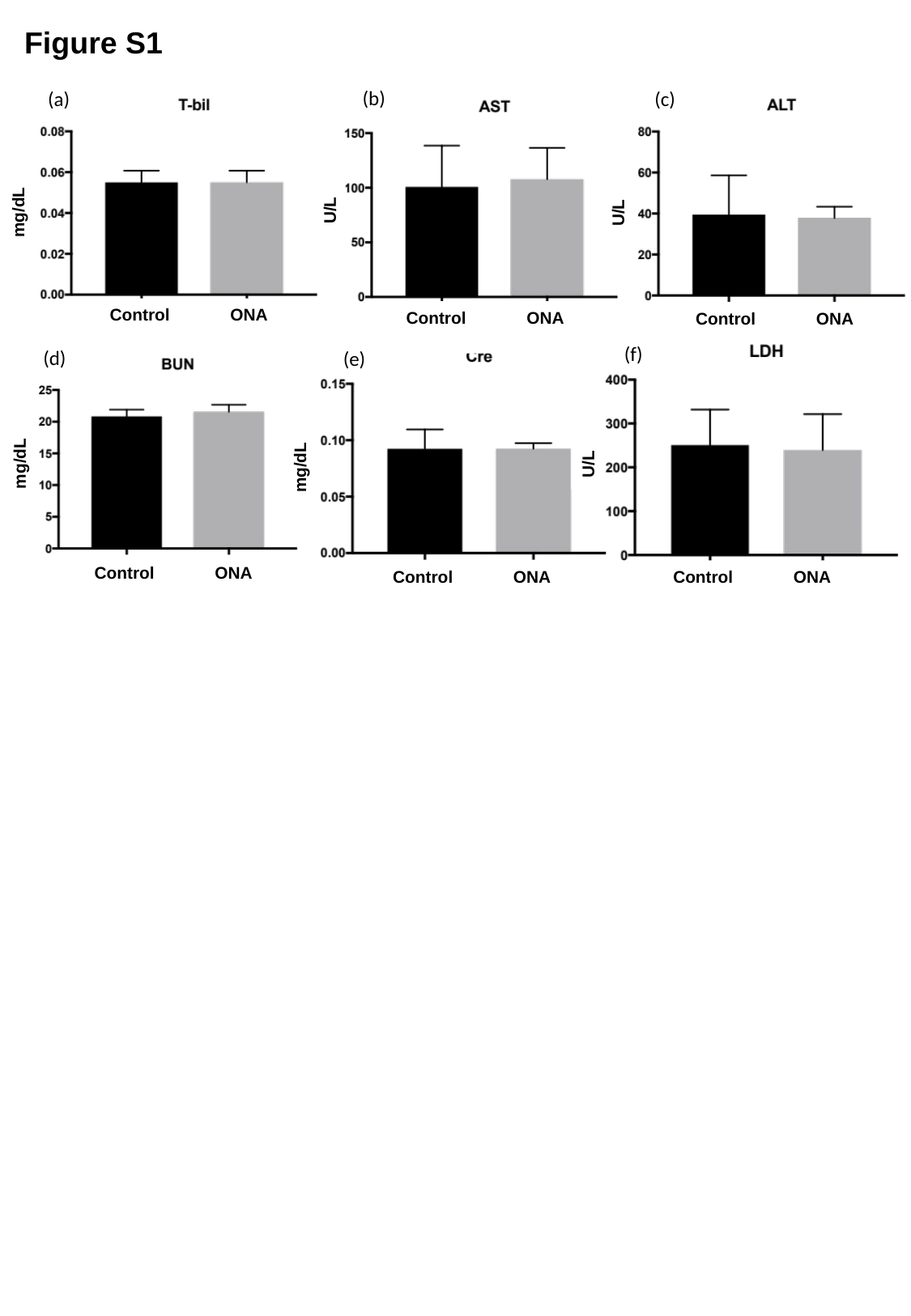

Figure S1
(b)
(a)
(c)
U/L
U/L
 mg/dL
 Control ONA
 Control ONA
 Control ONA
(f)
U/L
 Control ONA
(d)
(e)
 mg/dL
 mg/dL
 Control ONA
 Control ONA

## Slide 2
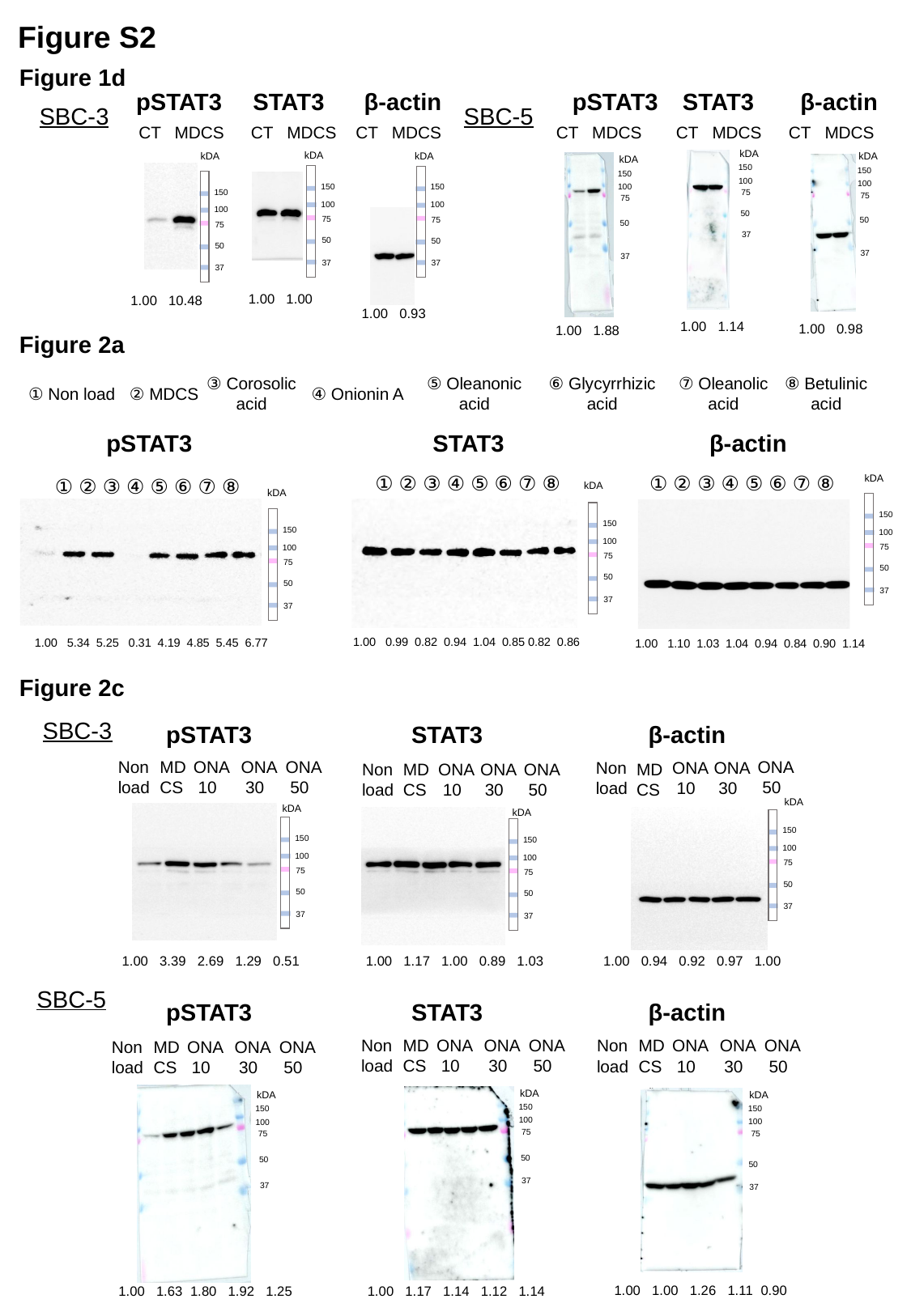

Figure S2
Figure 1d
pSTAT3
STAT3
β-actin
pSTAT3
STAT3
β-actin
SBC-5
SBC-3
CT MDCS
CT MDCS
CT MDCS
CT MDCS
CT MDCS
CT MDCS
kDA
150
100
75
50
37
kDA
kDA
kDA
kDA
150
100
75
50
37
kDA
150
100
75
50
37
150
100
75
50
37
150
100
75
50
37
150
100
75
50
37
1.00 1.00
1.00 10.48
1.00 0.93
1.00 1.14
1.00 0.98
1.00 1.88
Figure 2a
③ Corosolic
acid
⑤ Oleanonic
acid
⑥ Glycyrrhizic
acid
⑦ Oleanolic
acid
⑧ Betulinic
acid
① Non load
② MDCS
④ Onionin A
pSTAT3
STAT3
β-actin
① ② ③ ④ ⑤ ⑥ ⑦ ⑧
① ② ③ ④ ⑤ ⑥ ⑦ ⑧
kDA
① ② ③ ④ ⑤ ⑥ ⑦ ⑧
kDA
kDA
150
100
75
50
37
150
100
75
50
37
150
100
75
50
37
1.00 0.99 0.82 0.94 1.04 0.85 0.82 0.86
1.00 5.34 5.25 0.31 4.19 4.85 5.45 6.77
1.00 1.10 1.03 1.04 0.94 0.84 0.90 1.14
Figure 2c
SBC-3
pSTAT3
STAT3
β-actin
ONA
 50
Non
load
MDCS
ONA
 10
ONA
 30
ONA
 50
Non
load
ONA
 10
ONA
 30
MDCS
MDCS
Non
load
ONA
 10
ONA
 30
ONA
 50
kDA
kDA
kDA
150
100
75
50
37
150
100
75
50
37
150
100
75
50
37
1.00 3.39 2.69 1.29 0.51
1.00 1.17 1.00 0.89 1.03
1.00 0.94 0.92 0.97 1.00
SBC-5
pSTAT3
STAT3
β-actin
Non
load
MDCS
ONA
 10
ONA
 30
ONA
 50
Non
load
MDCS
ONA
 10
ONA
 30
ONA
 50
Non
load
MDCS
ONA
 10
ONA
 30
ONA
 50
kDA
150
100
75
50
37
kDA
150
100
75
50
37
kDA
150
100
75
50
37
1.00 1.00 1.26 1.11 0.90
1.00 1.63 1.80 1.92 1.25
1.00 1.17 1.14 1.12 1.14

## Slide 3
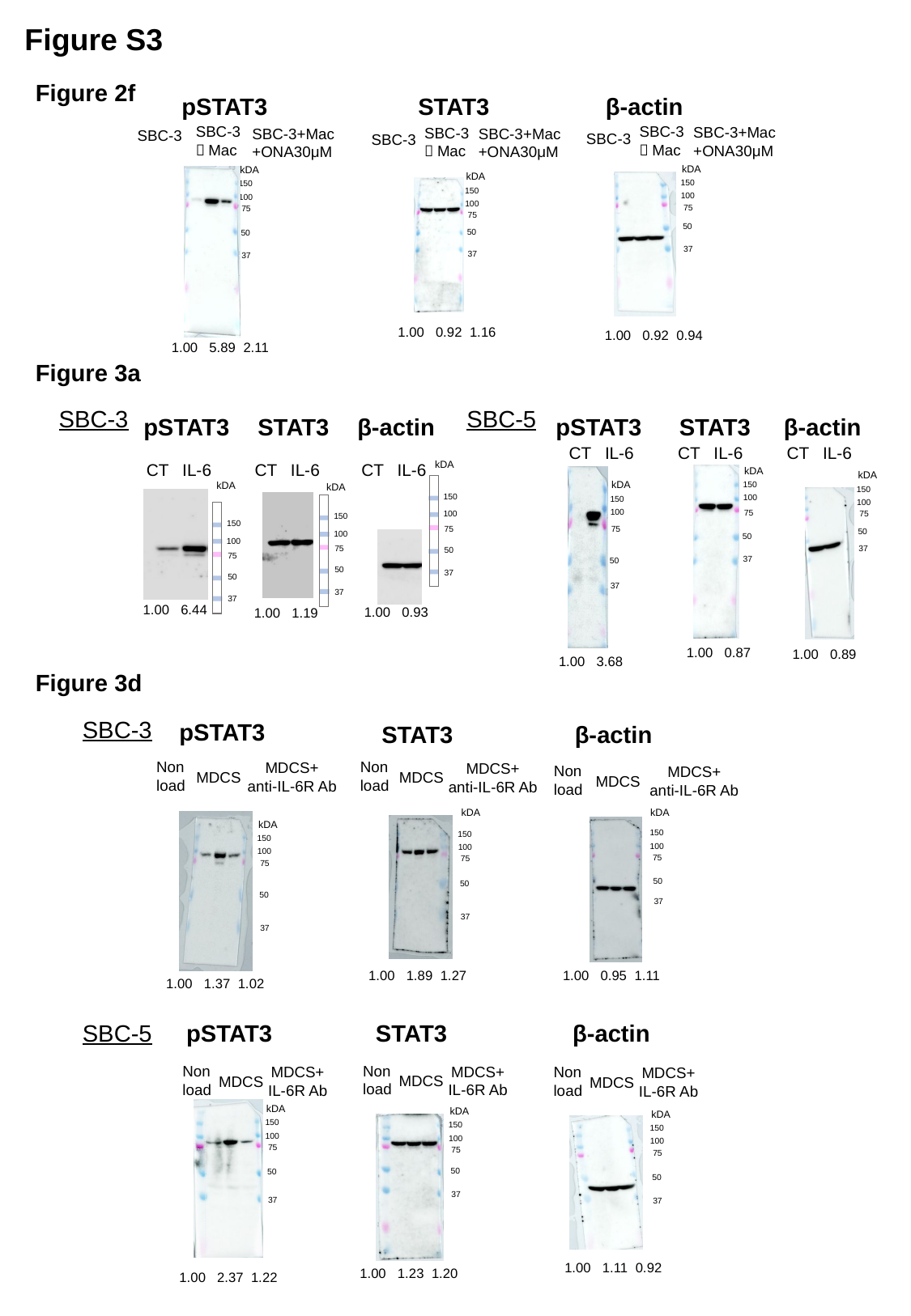

Figure S3
Figure 2f
pSTAT3
STAT3
β-actin
SBC-3
＋Mac
SBC-3
＋Mac
SBC-3+Mac
+ONA30μM
SBC-3
＋Mac
SBC-3+Mac
+ONA30μM
SBC-3+Mac
+ONA30μM
SBC-3
SBC-3
SBC-3
kDA
150
100
75
50
37
kDA
150
100
75
50
37
kDA
150
100
75
50
37
1.00 0.92 1.16
1.00 0.92 0.94
1.00 5.89 2.11
Figure 3a
SBC-3
SBC-5
pSTAT3
STAT3
β-actin
pSTAT3
STAT3
β-actin
CT IL-6
CT IL-6
CT IL-6
kDA
CT IL-6
CT IL-6
CT IL-6
kDA
150
100
75
50
37
kDA
150
100
75
50
37
kDA
150
100
75
50
37
kDA
kDA
150
100
75
50
37
150
100
75
50
37
150
100
75
50
37
1.00 6.44
1.00 0.93
1.00 1.19
1.00 0.87
1.00 0.89
1.00 3.68
Figure 3d
SBC-3
pSTAT3
STAT3
β-actin
Non
load
Non
load
MDCS+
anti-IL-6R Ab
MDCS+
anti-IL-6R Ab
Non
load
MDCS+
anti-IL-6R Ab
MDCS
MDCS
MDCS
kDA
kDA
kDA
150
100
75
50
37
150
150
100
100
75
75
50
50
37
37
1.00 1.89 1.27
1.00 0.95 1.11
1.00 1.37 1.02
SBC-5
pSTAT3
STAT3
β-actin
Non
load
Non
load
MDCS+
IL-6R Ab
Non
load
MDCS+
IL-6R Ab
MDCS+
IL-6R Ab
MDCS
MDCS
MDCS
kDA
150
100
75
50
37
kDA
150
100
75
50
37
kDA
150
100
75
50
37
1.00 1.11 0.92
1.00 1.23 1.20
1.00 2.37 1.22

## Slide 4
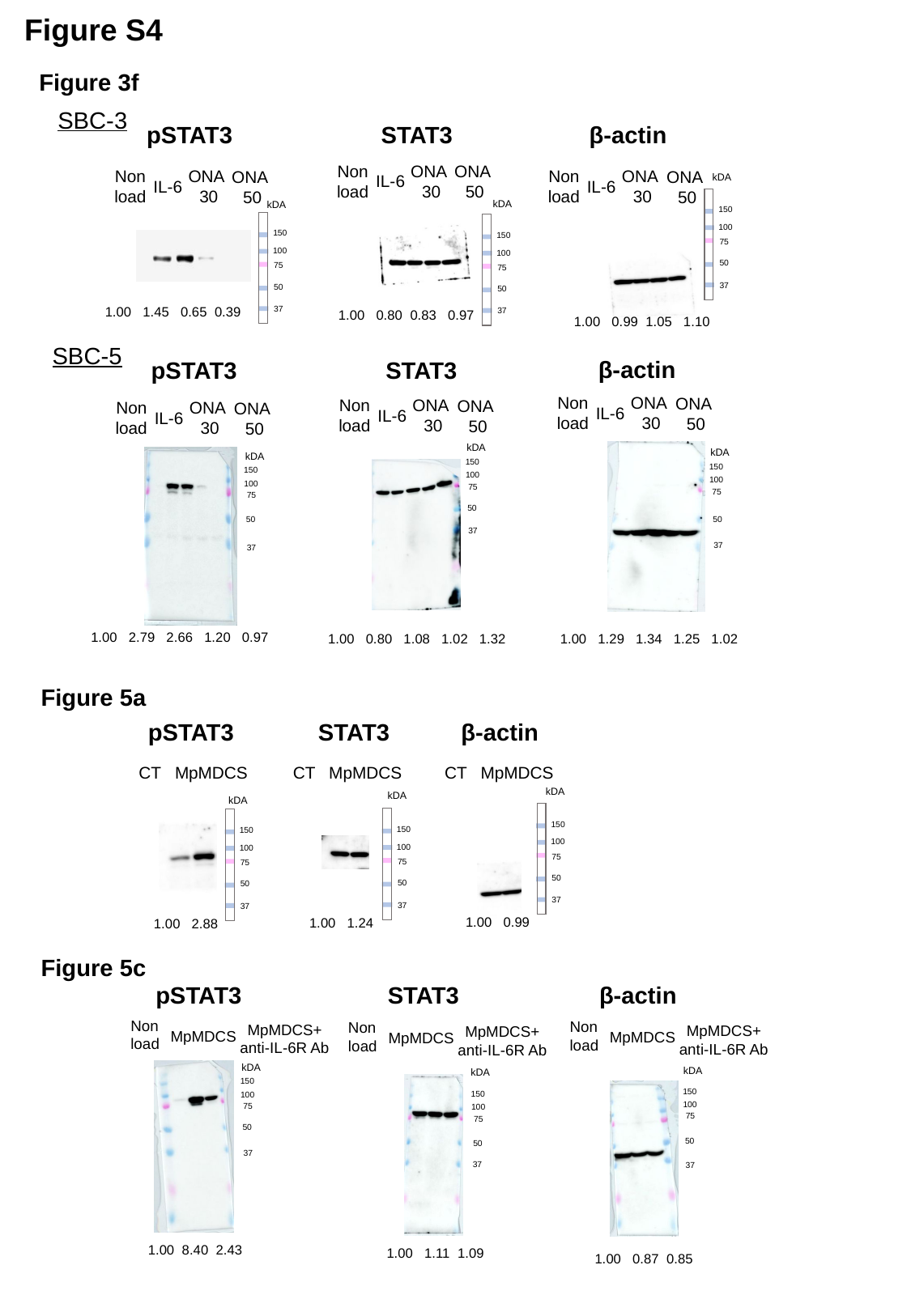

Figure S4
Figure 3f
SBC-3
pSTAT3
STAT3
β-actin
Non
load
ONA
 30
ONA
 50
Non
load
ONA
 30
Non
load
ONA
 30
ONA
 50
IL-6
ONA
 50
IL-6
kDA
IL-6
150
100
75
50
37
kDA
kDA
150
100
75
50
37
150
100
75
50
37
1.00 1.45 0.65 0.39
1.00 0.80 0.83 0.97
1.00 0.99 1.05 1.10
SBC-5
β-actin
pSTAT3
STAT3
Non
load
ONA
 30
ONA
 50
IL-6
Non
load
ONA
 30
ONA
 50
IL-6
Non
load
ONA
 30
ONA
 50
IL-6
kDA
150
100
75
50
37
kDA
150
100
75
50
37
kDA
150
100
75
50
37
1.00 2.79 2.66 1.20 0.97
1.00 0.80 1.08 1.02 1.32
1.00 1.29 1.34 1.25 1.02
Figure 5a
pSTAT3
STAT3
β-actin
CT MpMDCS
CT MpMDCS
CT MpMDCS
kDA
kDA
kDA
150
100
75
50
37
150
100
75
50
37
150
100
75
50
37
1.00 0.99
1.00 1.24
1.00 2.88
Figure 5c
pSTAT3
STAT3
β-actin
Non
load
Non
load
Non
load
MpMDCS+
anti-IL-6R Ab
MpMDCS+
anti-IL-6R Ab
MpMDCS+
anti-IL-6R Ab
MpMDCS
MpMDCS
MpMDCS
kDA
kDA
kDA
150
150
150
100
100
75
100
75
75
50
50
50
37
37
37
1.00 8.40 2.43
1.00 1.11 1.09
1.00 0.87 0.85
